# A case of anti-gamma-aminobutyric acid-B receptor encephalitis coexisting with ankylosing spondylitis

**DOI:** 10.1007/s10072-020-04336-2

**Published:** 2020-04-03

**Authors:** Aiqing Li, Weihua Feng, Jingfang Lin, Dong Zhou, Zhen Hong

**Affiliations:** 1 Department of Neurology, West China Hospital, Sichuan University, Chengdu, Sichuan People’s Republic of China; 2 Department of Experimental Medicine, West China Hospital, Sichuan University, Chengdu, Sichuan People’s Republic of China

Dear Editor,

Anti-gamma-aminobutyric acid-B receptor (GABA_B_-R) encephalitis, autoimmune encephalitis (AE) associated with anti-neuronal cell surface antibodies, presents with prominent seizures, behavioral changes, and cognitive deficits. Half of the cases of anti-GABA_B_-R encephalitis have been associated with tumors, especially small-cell lung cancer [1]. Ankylosing spondylitis (AS) is a chronic autoimmune inflammatory condition affecting the spine and sacroiliac joint [2], which leads to back pain and progressive spinal stiffness. However, the co-occurrence of anti-GABA_B_-R encephalitis and AS has never been reported. Here, we provide a case report of anti-GABA_B_-R encephalitis coexisting with AS for the first time. A Chinese man, whose HLA-B27 and anti-GABA_B_-R antibodies were positive, was diagnosed with anti-GABA_B_-R encephalitis and AS.

A 34-year-old man presented with his first generalized tonic-clonic seizure (GTCS) lasting for 1 min, 20 days before this admission. After a neurological evaluation and head computed tomography (CT) scan in the emergency department, he was prescribed sodium valproate 500 mg twice daily. However, he experienced another GTCS which lasted for 3 min, 20 days after his first GTCS. Other symptoms included psychiatric abnormalities, such as irritability, delirium, and behavioral changes, in addition to more frequent seizure attacks observed on the following days. The patient experienced GTCSs one-to-four times daily with each episode lasting for 1–3 min. He reported a history of lower back pain and fatigue for 2 years without any evaluation or treatment. An aunt of the patient had a history of ankylosing spondylitis. Neurological examination showed a decline in cognitive function, which mainly affected short-term memory, and disorientation in time and space was revealed. The results of cranial nerve (the first cranial nerve was not tested), motor system, tendon reflexes, meningeal irritation sign, and Babinski sign examinations were generally normal. It was not possible to completely examine the patient’s sensory system or motor coordination. GABA_B_-R antibodies were found in the serum (1:10) and cerebrospinal fluid (CSF) (1:10) using a Cytometric Bead Array (Fig. 1) (Euroimmun, Lubeck, Germany). Serum and CSF were both negative for antibodies against neuronal surface antigens, including antibodies to N-methyl-D-aspartate (NMDA) receptors, leucine-rich glioma-inactivated protein 1 (LGI1), contactin-associated protein-like 2 (CASPR2), α-amino-3-hydroxy-5-methyl-4-isoxazolepropionic acid receptor (AMPAR), dipeptidyl-peptidase-like protein-6 (DPPX), and metabotropic glutamate receptor 5 (mGluR5). Tests for paraneoplastic antibodies (anti-Hu, anti-Yo, anti-Ri, anti-GAD, anti-PCA2, anti-Ma2/Ta, anti-CV2/CRMP5, anti-ANNA-3, and anti-amphiphysin) in serum and CSF were negative. Anti-voltage-gated calcium channel (VGCC) antibodies (using radioimmunoprecipitation) were negative in serum, as well. The patient’s total protein level, glucose level, white blood count, and IgG synthetic rate of CSF specimen were unremarkable. No infectious etiology, such as bacteria, fungus, *Mycobacterium tuberculosis*, or cryptococcus, was detected in the CSF.

**Fig. 1:**
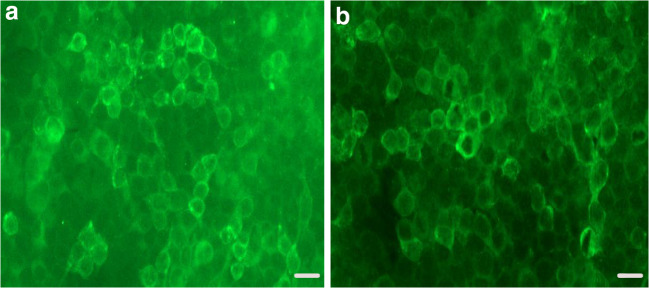
Antibody tests of the patient. **a** Cerebrospinal fluid showing binding to the surface of cells expressing anti-gamma-aminobutyric acid-B receptors (GABA_B_-Rs) (1:10). **b** Serum showing binding to the surface of cells expressing GABA_B_-Rs (1:10) (scale bar 10 μm)

Laboratory blood testing, including routine blood tests, routine biochemical evaluations, and tests for rheumatoid factors and rheumatological autoantibodies (anti-cyclic citrullinated peptides antibodies, anti-nuclear antibodies, anti-double-stranded DNA antibodies, anti-ENA autoantibody profile, and antiphospholipid antibodies); thyroid profile (triiodothyronine, thyroxine, thyroid-stimulating hormone); tumor biomarkers (prostate-specific antigen (PSA) and α-fetoprotein (AFP), carcinoembryonic antigen (CEA), carbohydrate antigen (CA)125, CA19-9, CA724); treponema pallidum; and viral serological tests (herpes simplex virus 1/2, human immunodeficiency virus, human cytomegalovirus, and Epstein-Barr virus) were all negative or normal, except for C-reactive protein, which was 25 mg/L (normal value < 5 mg/L). A genetic study revealed that the patient was HLA-B27-positive (flow cytometry) (Supplemental Fig. 1). CT scans of the abdomen and chest conducted upon this admission to detect an underlying tumor revealed no tumor signs. Fluid-attenuated inversion recovery and T1- and T2-weighted magnetic resonance imaging (MRI) of the patient’s brain were unremarkable (Supplemental Fig. 2 A–C). An electroencephalogram (EEG) documented a frontal slowing wave but without epileptic discharge (Supplemental Fig. 2 D–E). A spinal X-ray showed hyperosteogeny of the spine and narrowing of the lumbar intervertebral space with blurred space in the bilateral sacroiliac joints (Fig. 2). An MRI of the sacroiliac joints revealed narrow spaces between the bilateral sacroiliac joints with rough articular surfaces and abnormal signals below the articular surface, which indicated the possibility of AS. Additional laboratory results are shown in Table 1.

**Fig. 2:**
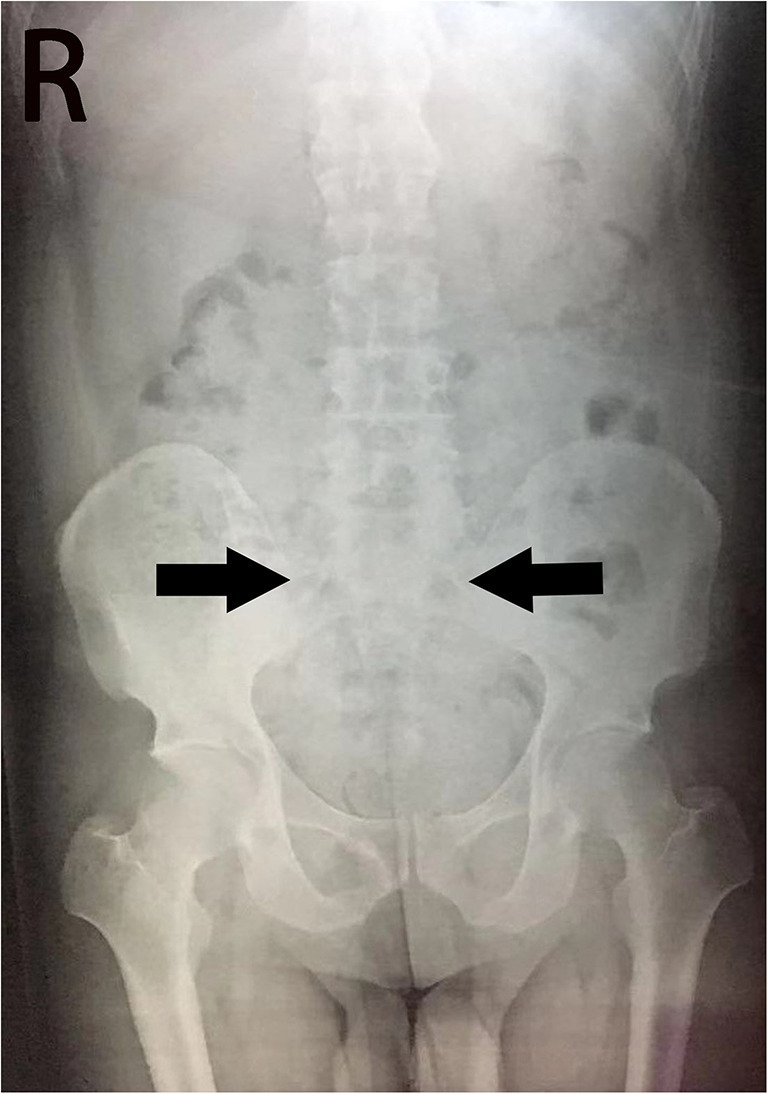
Spinal X-rays of the patient. Spinal x-rays show bilateral sacroiliitis with blurred articular surfaces (arrows), indicating the possibility of ankylosing spondylitis

**Table 1:** Results of laboratory examinations in the patient

Cerebrospinal fluid	(−)
Glucose	(−)
Protein	(−)
Fungus (smear/culture)	(−/−)
Bacteria (smear/culture)	(−/−)
Acid-fast bacilli cultures	(−)
Malignant cell	(−)
Flow cytometry	(−)
^a^Neuronal cell surface antibodies	(CSF/serum)
Anti-NMDAR	(−/−)
Anti-GABA_B_	(+/+)
Anti-LGI1	(−/−)
Anti-CASPR2	(−/−)
Anti-AMPAR	(−/−)
Anti-DPPX	(−/−)
Anti-mGluR5	(−/−)
^a^Onconeuronal antibodies	(CSF/serum)
Anti-Hu	(−/−)
Anti-Ri	(−/−)
Anti-Yo	(−/−)
Anti-GAD	(−/−)
Anti-PCA2	(−/−)
Anti-Ma2/Ta	(−/−)
Anti-CV2/CRMP5	(−/−)
Anti-ANNA3	(−/−)
Anti-amphiphysin	(−/−)
^b^Anti-VGCC antibodies	(−)
Hematologic test results	
HLA-B27 gene	(+)
Human cytomegalovirus	(−)
Epstein-Barr virus	(−)
Human immunodeficiency virus	(−)
Syphilis	(−)
Mycobacteria	(−)
Prostate-specific antigen	(−)
α-fetoprotein	(−)
Carcinoembryonic antigen	(−)
Carbohydrate antigen 125	(−)
Carbohydrate antigen 19-9	(−)
Carbohydrate antigen 724	(−)
Anti-nuclear antibody	(−)
Anti-keratin antibodies	(−)
Anti-dsDNA	(−)
Anti-Smith antibodies	(−)
Anti-SS-A and SS-B	(−)
Anti-ANCA	(−)
Anti-anticardiolipin antibodies	(−)
Rheumatoid factor	(−)
Anti-CCP	(−)
Anti-topoisomerase antibody	(−)
Anti-Jo-1	(−)
Anti-RNP-70	(−)
Anti-centromere B	(−)
Anti-streptolysin O	(−)
C-reactive protein	25 mg/L (normal value < 5 mg/L)
Erythrocyte sedimentation rate	(−)
Triiodothyronine	(−)
Thyroxine	(−)
Free triiodothyronine	(−)
Free thyroxine	(−)
Thyroid-stimulating hormone	(−)
Thyroid globulin antibody	(−)
Thyroid peroxidase antibody	(−)

^a^Cell base assay (EUROIMMUN, Germany)

^b^Radioimmunoprecipitation (EUROIMMUN, Germany)

N-methyl-D-aspartate receptor, *NMDAR*; gamma-aminobutyric acid-B receptor, *GABAB*; leucine-rich glioma-inactivated protein 1, *LGI1*; contactin-associated protein-like 2, *CASPR2*; α-amino-3-hydroxy-5-methyl-4-isoxazolepropionic acid receptor, *AMPAR*; dipeptidyl-peptidase-like protein-6, *DPPX*; metabotropic glutamate receptor 5, *mGluR5*; anti-neuronal nuclear antibodies type 1, *anti-Hu*; anti-neuronal nuclear antibodies type 2, *anti-Ri*; anti-cytoplasmic purkinje cell antibodies, *Anti-Yo*; anti-glutamic acid decarboxylase, *anti-GAD*; anti-cytoplasmic purkinje cell antibodies, *anti-PCA2*; collapsing response mediator protein 5, *CRMP5*; anti-neuronal nuclear antibodies type 3, *ANNA3*; anti-voltage-gated calcium channel, *VGCC*; human leukocyte antigen, *HLA*; gene double-stranded deoxyribonucleic acid, *dsDNA*; anti-SS-A(Ro) and anti-SS-B(La) autoantibodies, *Anti-SS-A* and *SS-B*; anti-neutrophil cytoplasmic antibodies, *anti-ANCA*; cyclic peptide containing citrulline, *CCP*; anti-aminoacyl tRNA synthetase antibodies, *anti-Jo-1*; anti-recombinant 70 kDa ribonucleoprotein, *anti-RNP-70*

Immune therapy (intravenous methylprednisolone, 1000 mg/day for 5 days), accompanied by oral valproate and oxcarbazepine, was initiated immediately after the definite diagnosis 27 days after onset. Then, oral prednisolone was started at 60 mg/day and was reduced by 5 mg weekly. The response to immunotherapy and the combination of anti-epileptic drugs were reasonably good, as indicated by the absence of seizures. The cognitive impairments and behavior abnormalities improved significantly after 4 weeks of immune therapy. Stiffness and pain in the lower back persisted because the patient did not accept non-steroidal anti-inflammatory drugs that could have alleviated the discomfort. The GABA_B_-R antibodies were measured again after 1 month of immune therapy and showed negative results in both the CSF and serum. Chest and abdominal CT scans were performed, revealing no tumor signs 6 months and 1 year after disease onset. The patient’s symptoms resolved, and he reported no additional seizure episodes upon follow-up.

## Discussion

Based on the seizures and the abnormal psychiatric symptoms, in addition to the evidence of GABA_B_-R antibodies detected in the CSF and serum, the patient was diagnosed with anti-GABA_B_-R encephalitis. Our case also met the diagnostic criteria for AS by the positive HLA-B27 test and manifestations on spine and sacroiliac joint imaging. Reports on the co-occurrence of anti-GABA_B_-R encephalitis with tumors or another paraneoplastic neurologic disorder, such as Lambert-Eaton myasthenic syndrome, have been published [3]. However, a literature search for reports on anti-GABA_B_-R encephalitis or AE appearing with AS yielded nothing. Likewise, there were no reports of investigations undertaken to explain the relationship between anti-GABA_B_-R encephalitis and AE with AS. Some research has suggested that T cell-based autoimmunity plays an important role in the development of anti-GABA_B_-R encephalitis [4]. Similarly, T cells were found to act in key pathogenetic roles in AS, as well [2]. In addition, interleukin 17 (IL-17) plays a key role in inflammatory pathways and there is an evidence that IL-17 receptor-deficient mice may be protected against experimental AE. Furthermore, inflammatory disorders have also been associated with IL-17, and therapeutically targeting this inflammatory pathway could improve the outcome of AS patients because similar inflammatory pathways may exist between the two diseases [5].

Patients with anti-gamma-aminobutyric acid-B receptor encephalitis are frequently found to have coexisting small-cell lung cancer and some other types of tumors. Abdominal and chest CT scans performed on this patient upon admission revealed no tumor signs. However, further imaging examinations should be performed in other organs (for example, PET-CT). Unfortunately, our patient refused to undergo a PET-CT scan due to economic reasons, which limited the screening and detection of other potential tumors.

To better understand the coexistence of both diseases and to improve the prognosis, larger epidemiological studies and more research on the pathogenesis are warranted.

## Electronic supplementary material

Supplementary Fig. 1 HLA-B27 antigen of the patient. (1.A-C) Positive HLA-B27 antigen are showed on the surface of CD3+T lymphocytes by flow cytometry (FCM): (1.A) Lymphocyte are selected (arrow); (1.B) CD3+T lymphocytes are selected (arrow); (1.C) HLA-B27 monoclonal antibody was combined with HLA-B27 antigen on the surface of CD3+T lymphocytes. The software (provided by BD FACSCanto) calculated the mean fluorescence intensity of cell anti HLA-B27FL1 signal (arrow), it is greater than the threshold value set by the instrument (BD FACSCanto), it is reported as HLA-B27 positive. (PNG 4468 kb)

High resolution image (TIF 1705 kb)

Supplementary Fig. 2 Brain magnetic resonance imaging and electroencephalogram of the patient. (2.A-C) Magnetic Resonance imaging (MRI) of the brain were unremarkable: (2.A): T1-weighted of MRI; (2.B): T2-weight of MRI; (2.C): Fluid-attenuated inversion recovery weight of MRI (2.D-E) Electroencephalogram (EEG) of the patient: (2.D) showed frontal slowing wave but without epileptic discharge; (2.E) showed normal after immunotherapy. (PNG 13221 kb)

High resolution image (TIF 29710 kb)

## Data Availability

Further anonymized data can be made available to qualified investigators upon request to the corresponding author.
