# Supplementary figures and images for: A case of anti-gamma-aminobutyric acid-B receptor encephalitis coexisting with ankylosing spondylitis
Source: Neurol Sci. 2020 Apr 3;41(9):2631–4. doi: 10.1007/s10072-020-04336-2 (PMC7419346; doi:10.1007/s10072-020-04336-2)

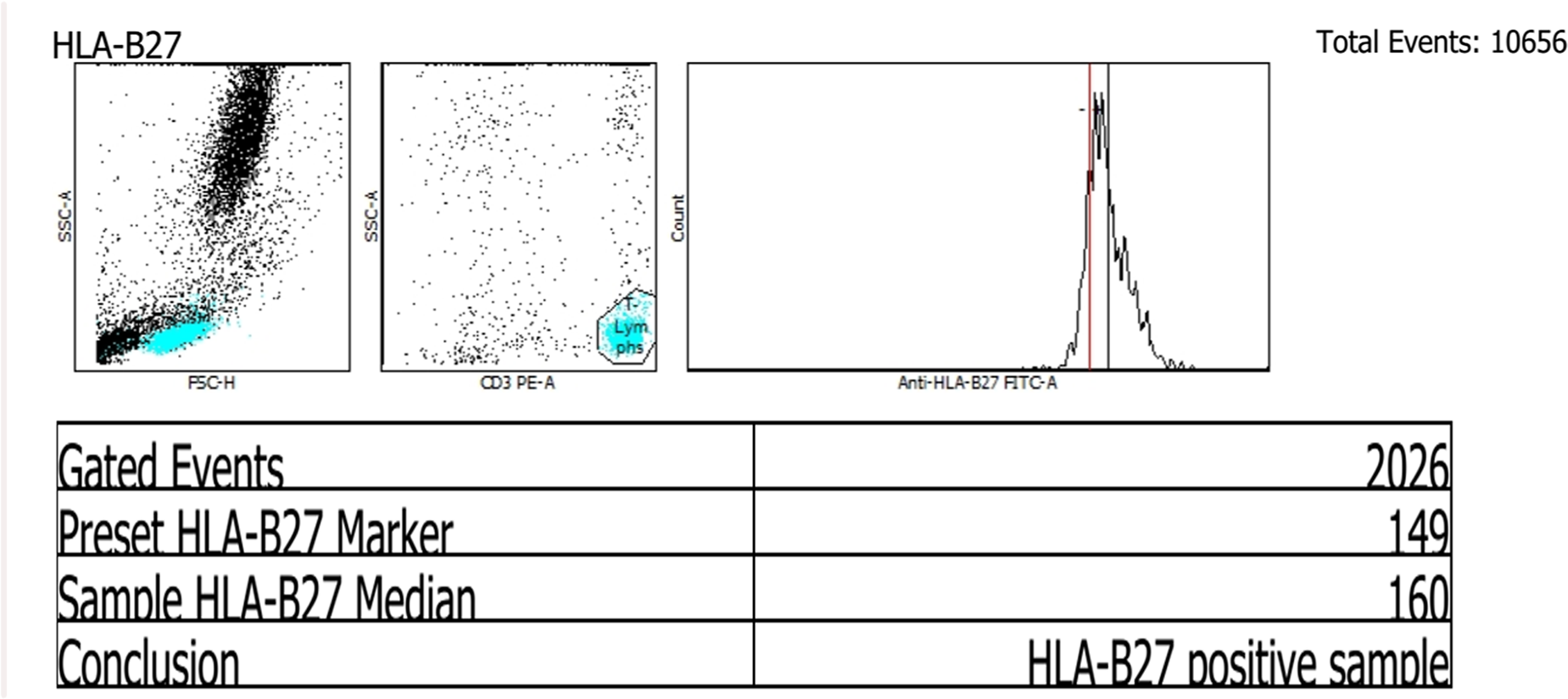

Supplement: Supplementary file 1 — HLA-B27 antigen of the patient. (1.A-C) Positive HLA-B27 antigen are showed on the surface of CD3+T lymphocytes by flow cytometry (FCM): (1.A) Lymphocyte are selected (arrow); (1.B) CD3+T lymphocytes are selected (arrow); (1.C) HLA-B27 monoclonal antibody was combined with HLA-B27 antigen on the surface of CD3+T lymphocytes. The software (provided by BD FACSCanto) calculated the mean fluorescence intensity of cell anti HLA-B27FL1 signal (arrow), it is greater than the threshold value set by the instrument (BD FACSCanto), it is reported as HLA-B27 positive. (PNG 4468 kb) [file 10072_2020_4336_Fig3_ESM.png]

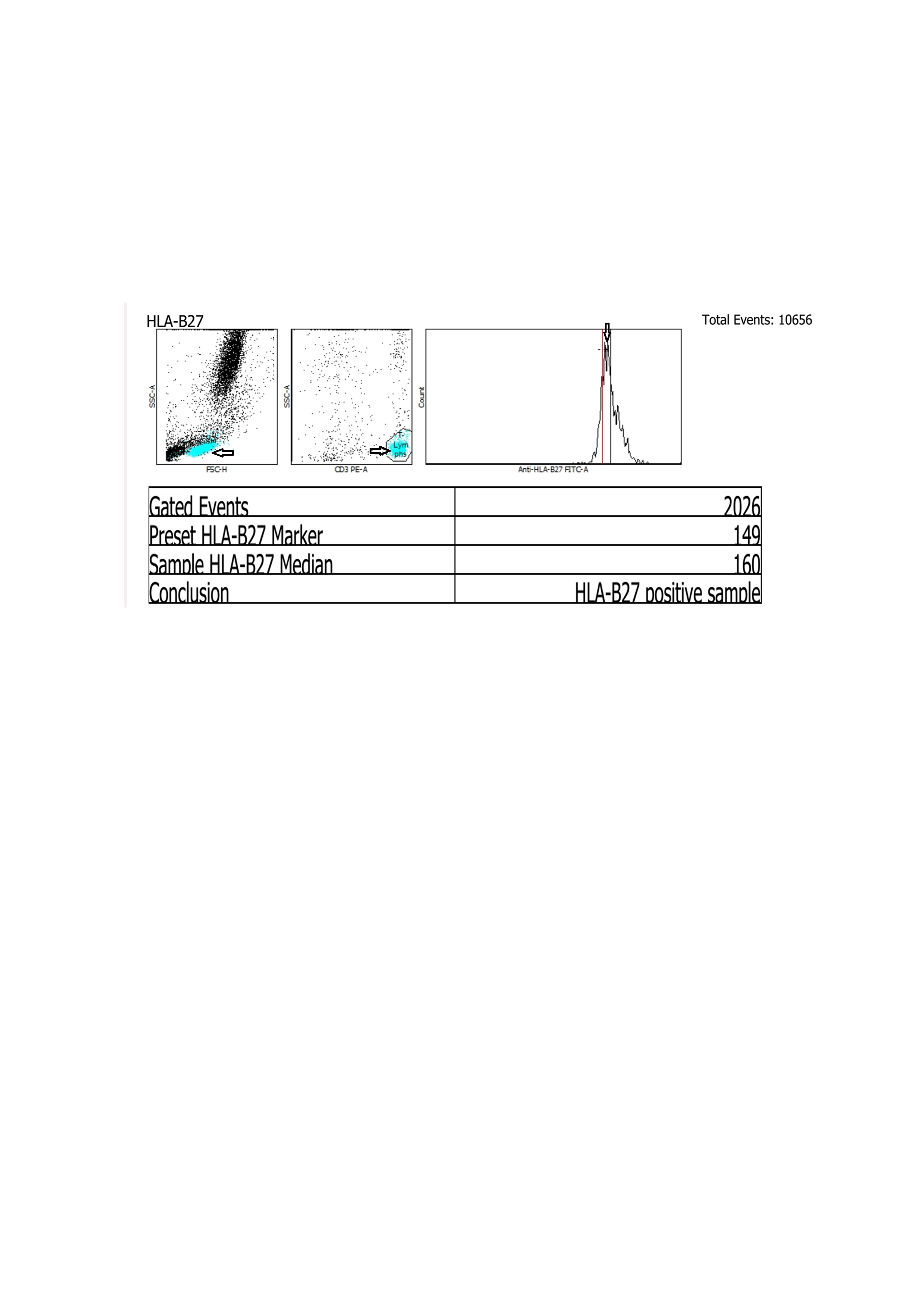

Supplement: Supplementary file 2 — High resolution image (TIF 1705 kb) [file 10072_2020_4336_MOESM1_ESM.tif]

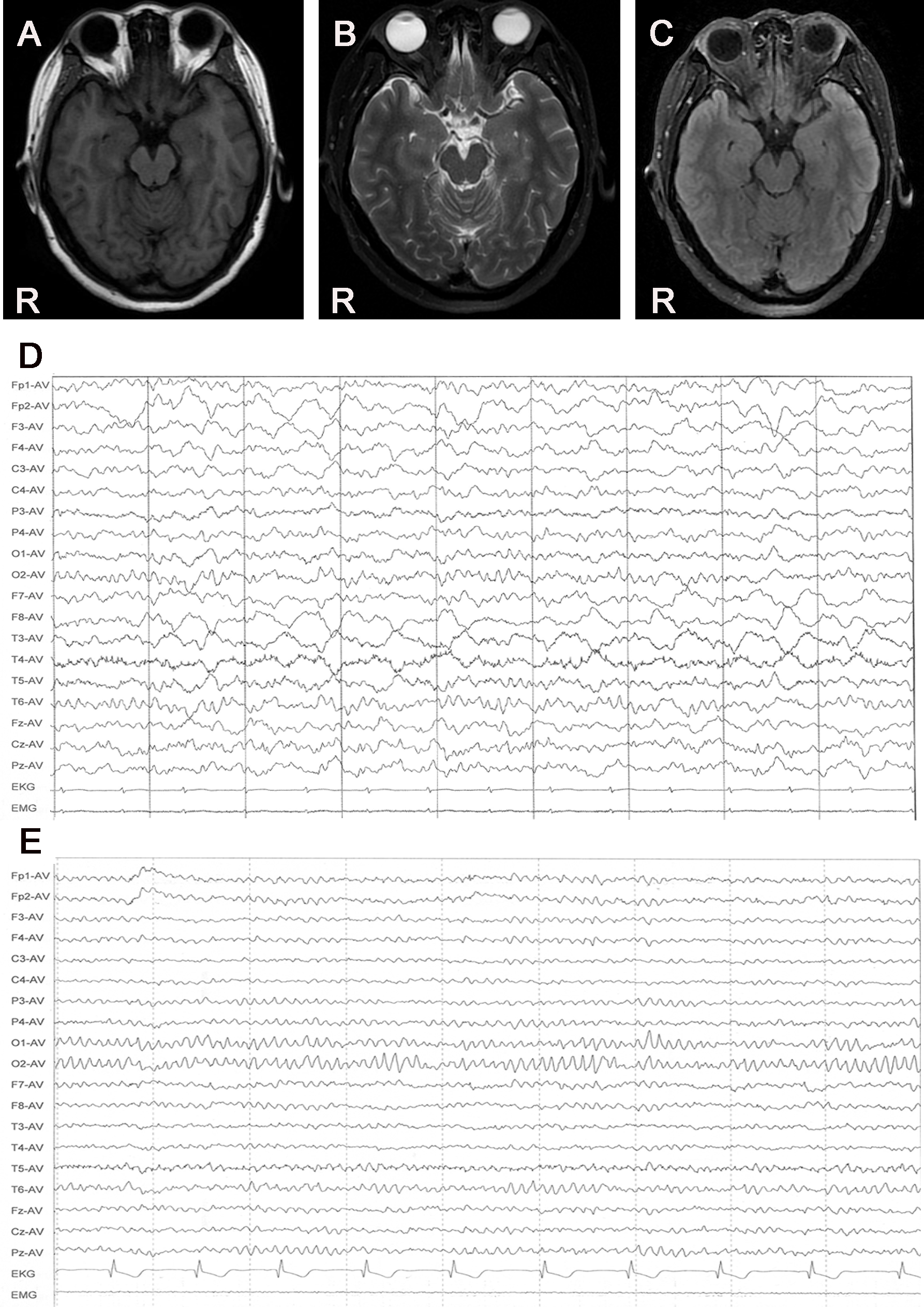

Supplement: Supplementary file 3 — Brain magnetic resonance imaging and electroencephalogram of the patient. (2.A-C) Magnetic Resonance imaging (MRI) of the brain were unremarkable: (2.A): T1-weighted of MRI; (2.B): T2-weight of MRI; (2.C): Fluid-attenuated inversion recovery weight of MRI (2.D-E) Electroencephalogram (EEG) of the patient: (2.D) showed frontal slowing wave but without epileptic discharge; (2.E) showed normal after immunotherapy. (PNG 13221 kb) [file 10072_2020_4336_Fig4_ESM.png]

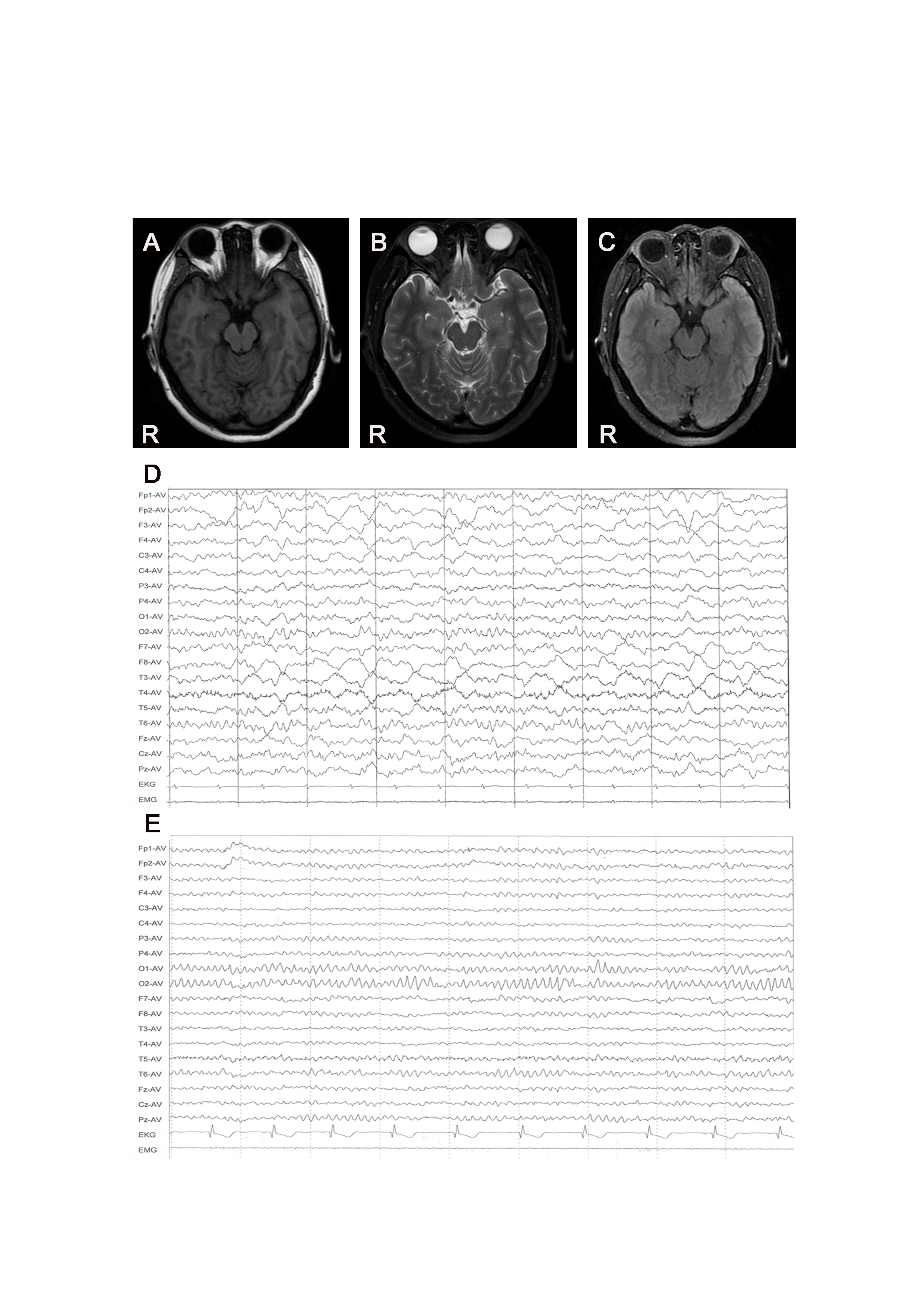

Supplement: Supplementary file 4 — High resolution image (TIF 29710 kb) [file 10072_2020_4336_MOESM2_ESM.tif]
